# Refined methodologies for probabilistic dietary exposure assessment for food contaminants based on the observed individual means methodology

**DOI:** 10.1038/s41370-024-00740-4

**Published:** 2025-01-29

**Authors:** Simone Stefano, Alessia Lanno, Sofia Ghironi, Alice Passoni, Renzo Bagnati, Alessandra Roncaglioni, Enrico Davoli, Elena Fattore

**Affiliations:** Department of Environmental Health Sciences, Istituto di Ricerche Farmacologiche Mario Negri IRCCS, Via Mario Negri 2, 20156 Milano, Italy

**Keywords:** Risk Assessment, Exposure Assessment, Observed Individual Means, Stratified non-parametric bootstrap, 3-monochloropropane-1,2-diol

## Abstract

**Background:**

The Observed Individual Means (OIM) methodology, based on the non-parametric bootstrap, is usually employed to perform basic probabilistic dietary chronic exposure assessment, and assumes independence and identical distribution of occurrence data within food category. However, this assumption may not be valid if several expected distributions of occurrence can be a priori identified within food category. Moreover, OIM assumes each analysed food sample to equally contribute to mean occurrence, as information about relevance of each food item cannot be incorporated into exposure assessment.

**Objective:**

In this paper we address the above-mentioned violations and develop two statistical methodologies to accommodate for them into OIM.

**Methods:**

The stratified non-parametric bootstrap and weighted mean occurrence are employed to correct for such violations. As a case study, we compare the methodologies by estimating the exposure of the adult Italian population to the process contaminant 3-monochloropropane-1,2-diol.

**Results:**

We propose strategies to interpret their results and show their relevance in conducting exposure assessment.

**Impact statement:**

For the first time in the literature, we critically examine a widely used methodology for Probabilistic Dietary Exposure Assessment from a statistical perspective, focusing on the underlying assumptions and their potential violations in real-world scenarios. We then develop techniques to address these violations, providing a more accurate and robust approach to exposure assessment. This work is particularly relevant for risk assessors and managers, since it offers a refined toolset for more precise exposure assessments.

## Introduction

The Observed Individual Means (OIM) methodology is recommended by the European Food Safety Authority (EFSA) for conducting chronic dietary exposure assessments [1]. From a statistical point of view, OIM is part of the Probabilistic Exposure Assessment (PEA) framework, as it jointly accounts for variability and uncertainty [2]. OIM is used as basic, first-tier methodology, or as replacement for more complex methodologies, such as the BetaBinomial-Normal or the Logistic Normal-Normal models. Its key theoretical drawbacks involve the lack of separation between intra- and inter-individual variation and the inability to handle covariates. However, it maintains its popularity on the grounds of its simplicity, easiness of interpretability, and inherent conservativeness of upper percentiles, which prevents from setting too low safety limits [3]. Nonetheless, little statistical formalization, guidance, and exemplification regarding this methodology is provided in literature, making this simple non-parametric procedure potentially hard to master for professionals not acquainted with resampling procedures.

An often overlooked aspect of OIM is that exposure is computed as a function of food category-specific mean occurrences of contaminants. OIM relies on the non-parametric bootstrap [4], which assumes statistical units to be independent and identically distributed (i.i.d.), implying random sampling of occurrence data. However, this is rarely achievable in practice. For example, the assumption of identical distribution is violated if subgroups of food items can be clearly a priori identified within category according to their expected level of contamination. In that case, the distribution of occurrence should be regarded as a mixture of random variables. Another relevant aspect is that OIM implicitly assumes equal contribution of each analysed food product to mean occurrence. Therefore, information about consumption of different products cannot be readily incorporated to compute more realistic exposure estimates. To generate more refined exposure estimates, both above-mentioned features should be considered.

The advantage of implicitly modelling subcategories within the same category concerns the readability of an output of exposure assessment, namely the investigation of the contribution of individual foods to the higher intake [1]. In the case of contaminants present in various categories, risk assessors may want to provide an overview on a category level rather than on a subcategory level to avoid fragmentation of the output. This could especially benefit exposure assessment for widespread contaminants, as for example cadmium [5] and mercury [6].

Sensitivity analyses constitute relevant tools in the assessment of the robustness of assumptions made for the primary exposure assessment. Sources of uncertainty are related to occurrence data, consumption data, and modeling [7]. Moreover, the increased availability of raw occurrence and consumption data repositories may foster the implementation of algorithms serving as sensitivity analyses for OIM. To the best of our knowledge, none of this kind has been proposed in literature and implemented in PEA-dedicated toolboxes employed by risk assessors and managers, as Monte Carlo Risk Assessment (MCRA) [3, 8].

In this article, we define and present two new methodologies, which were designed as sensitivity analyses for OIM, but can also be considered as standalone techniques. The stratified Observed Individual Means methodology (sOIM) addresses the violation of the i.i.d. assumption by employing the stratified bootstrap. On top of that, the weighted stratified Observed Individual Means methodology (wsOIM) also addresses the violation of the assumption of equal contribution of each food product to mean occurrence by computing a weighted mean occurrence.

The proposed methodologies are employed to estimate the exposure of the adult Italian population to 3-monochloropropane-1,2-diol (3-MCPD) via vegetable oils. 3-MCPD is a food-borne contaminant formed during the high-temperature refinement of edible oils, particularly during deodorization [9–11]. Currently, it is a widespread contaminant found in refined vegetable oils and various food items such as cookies, salted crackers, rusks, bread, and infant formulas [12, 13]. In literature, speculations have been made about different expected distributions of 3-MCPD occurrence in various types of vegetable oil [14], suggesting that the i.i.d. assumption may be violated within this category. Moreover, it would be pertinent to address the higher consumption of olive oil by the Italian population [15] in estimating exposure.

Risk characterization of the findings of our case study is carried out, given that EFSA has established a Tolerable Daily Intake of 2 μg/kg bw/day for 3-MCPD [16].

In this paper we used data on occurrence of 3-MCPD in vegetable oils gathered in the framework of our recent analyses of food samples (funded by the Italian Ministry of Health; grant number: RF-2019-12369154) to demonstrate the proposed methodologies.

## Methods

Given a food classification hierarchy - as for example the FoodEx2 Exposure Hierarchy [17] - let the term category identify food groups on the same hierarchical level, and the term subcategory identify food groups hierarchically nested within a parent category.

As a preliminary consideration, the original OIM methodology requires classification of food items to match between consumption and occurrence data. OIM is based on the propagation of uncertainty to exposure through the bootstrap [4], which is applied independently to each dataset. Individuals from a dietary survey are sampled with replacement to mimic uncertainty in the variability of daily consumption and body weight distributions. Separately, occurrence data are sampled with replacement to mimic uncertainty in mean occurrence. Body weight and category-specific daily consumption from the resampled consumers are then combined with the respective category-specific bootstrap mean occurrence. Iterating this procedure leads to the calculation of Confidence Intervals (CIs) – also named Probabilistic Estimates (PEs) - through the percentile method.

### sOIM and wsOIM methodologies

sOIM and wsOIM are slight modifications of OIM with respect to the application of the bootstrap to occurrence data. sOIM and wsOIM can be implemented only if additional information about subcategory are available in the occurrence data. For both, the standard bootstrap is replaced by the stratified bootstrap. Occurrence data are resampled within each subcategory: the sample size of each subcategory in the bootstrapped samples is being controlled [18], effectively addressing the violation of the i.i.d. assumption [19].

Regarding sOIM, further steps for exposure estimation remain unchanged compared to OIM.

wsOIM modifies sOIM by calculating a weighted mean occurrence - instead of the arithmetic mean-, given a vector of weights.

If consumption data indicate subcategories on top of categories, wsOIM can be used to account for the actual consumption of items belonging to each subcategory, in a consumption-aware approach (wsOIM*). The choice of weights should be based on each subcategory’s relative contribution to daily consumption of the category. wsOIM can also be used in a sensitivity analysis approach (wsOIM°): hypothetical scenarios can be constructed, in which weighting is used to investigate the variations of exposure estimates if occurrence values related to a specific subcategory – or specific subcategories – are allowed to contribute to exposure to greater extent. Therefore, the aim of wsOIM° is to assess the robustness of the assumption of equal contribution or of the weighting scheme employed for wsOIM*. In this latter case, the vectors of weights for wsOIM° should at least be more extreme than the one used for wsOIM*. In any case, weighting schemes for wsOIM° should be established so that more imbalanced consumption-aware weighting schemes should be regarded as implausible. wsOIM° was inspired by the Tipping Point methodology to assess the robustness of the assumption for missing data imputation in the clinical trials setting [20].

**Fig. 1:**
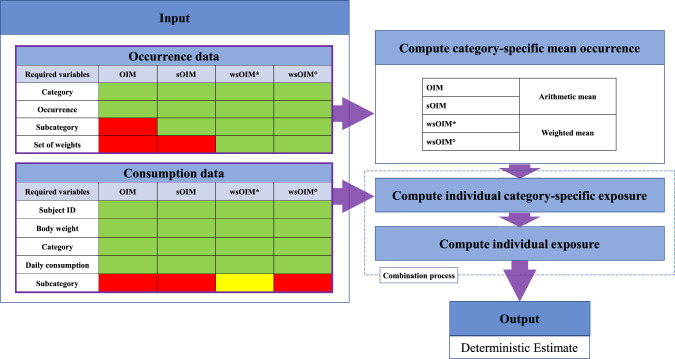
Flowchart for the computation of Deterministic Estimates. To apply the methodologies, different sets of variables should be available to the user; indications are reported in the “Input” step and presented separately for occurrence and consumption data. Variables depicted in green are essential for the application of the selected methodology. Variables depicted in red are not required. Variables depicted in yellow should preferentially be present. Specifications about the computation of mean occurrence employing either the arithmetic or a weighted mean are reported in the “Compute category-specific mean occurrence” step.

**Fig. 2:**
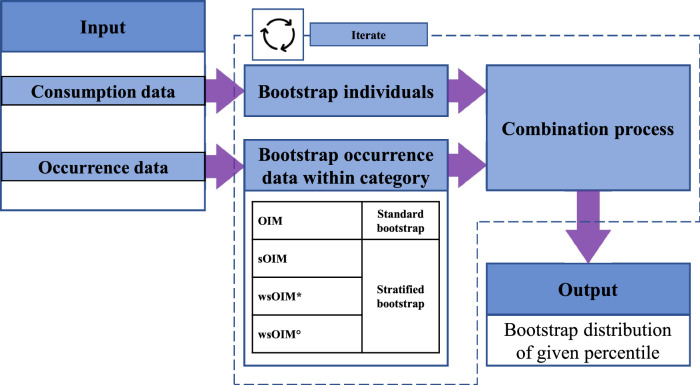
Flowchart for the computation of Probabilistic Estimates. Specifications about the application of either the standard or the stratified bootstrap are reported in the “Bootstrap occurrence data within category” step. Refer to Fig. 1 for specifications about the “Combination process”.

Workflows for the computation of Deterministic Estimates (DEs) and PEs are outlined in Fig. 1 and Supplementary Methods 1, and Fig. 2 and Supplementary Methods 2.

#### Simulation study

The performances of OIM, sOIM and wsOIM in estimating the exposure of the adult Italian population to 3-MCPD were compared through a simulation study in R [21]. For clarity, the vegetable oil (VO) category was considered as the only category of interest. Expansion to multiple categories is beyond the scope of this paper.

##### Food consumption data: preliminary operations

The publicly available data from the *INRAN SCAI 2005-2006* food consumption survey [15] were downloaded from the Food and Agriculture Organization/World Health Organization Global Individual Food consumption data Tool (*FAO/WHO GIFT*) web portal [22]. The data consisted of two datasets: one containing consumption information (dataset *C1*) and another containing demographic information (dataset *C2*). This food diary-based survey targeting the Italian population recorded food consumption for 3323 participants from 1329 households randomly selected from the telephone directory.

Based on the FoodEx2 codes [17] and plain text food descriptions provided by the investigators, only records in dataset *C1* pertaining to vegetable oil were retained for further analysis. Fortified olive and corn oils were recoded to their equivalent non-fortified forms. Ultimately, following the classification system used by the dietary survey investigators, nine types of vegetable oils were observed to be consumed: olive oil, corn oil, flaxseed oil, mixed seeds oil, peanut oil, rice oil, soybean oil, sunflower oil and extra virgin olive oil. The overall daily consumption of vegetable oil for each individual was computed.

One individual was discarded due to missing body weight information, resulting in 2830 individuals aged 18 years or older being included in the case study.

##### Occurrence dataset: preliminary operations

Fourteen samples of seed oil products (corn oil: 3 samples, mixed seeds oil: 2 samples, peanut oil: 3 samples, rice oil: 1 sample, soybean oil: 1 sample, sunflower oil: 4 samples) were analysed using a non-direct gas chromatography-mass spectrometry technique [23, 24]. A non-probabilistic convenience sampling approach was employed to collect samples from two major Italian supermarkets located in Lombardy, in October 2022. The choice of types of vegetable oils to be analysed was based on the results of the dietary survey. Products from the most renowned companies were sampled without replacement for each subcategory.

##### Methodology

Three subcategories were identified: Seed Oils (SO), Olive Oil (OO), and Extra-Virgin Olive Oil (EVOO). Consequently, SO was assumed to account for the consumption and occurrence of corn oil, flaxseed oil, mixed seeds oil, peanut oil, rice oil, soybean oil, and sunflower oil.

The simulation study was set up as follows.

Firstly, 1000 bootstrap samples of consumers were obtained from the consumption data.

Secondly, censored normal, lognormal, gamma and Weibull distributions were fitted to the values of occurrence related to SO via the *fitdistrplus* package [25]. These were chosen since defined in the positive real numbers. Goodness of fit was evaluated through the Akaike Information Criterion (AIC). The lognormal distribution defined by the parameter estimates $\hat{{{\rm{\mu }}}}=-1.48$, $\hat{{{\rm{\sigma }}}}=1.20$ showed the lowest AIC, thus the best fit (AICs for each distribution are reported in Supplementary Methods 3). Occurrence data were simulated from three different distributions to mimic the violation of i.i.d. assumption. For each vector of weights, 1000 occurrence datasets were obtained, each composed of 3000 occurrence values, i.e. 1000 for each subcategory. Occurrence values for SO were generated as ${C}_{{SO}}\sim {lognorm}(\hat{{{\rm{\mu }}}}{{;}}1)$. Vegetable oils produced from pulp fruits are expected to be more contaminated than vegetable oils produced from seeds because of the higher water content [14]: occurrence values for OO were therefore generated as ${C}_{{OO}}\sim {lognorm}({{\rm{\alpha }}}{{;}}1)$ where ${{\rm{\alpha }}}=\log (1.5+\exp (\hat{{{\rm{\mu }}}}))$, i.e. its median was 1.5 μg/g higher than the median of *C*_*SO*_. Contamination is expected to be negligible for EVOO since it is not refined, and given LOD=0.035 μg/g, occurrence values for EVOO were generated as ${C}_{{EVOO}} \sim {lognorm}(\log (0.035){{;}}1)$, i.e., half of the generated values was below LOD. To implement a middle-bound scenario for left-censored data, any occurrence below LOD was set to $\frac{{{\rm{LOD}}}}{2}$.

Thirdly, OIM, sOIM and wsOIM were applied to each simulated occurrence dataset via the *boot* package [26]. Three weighting schemes were generated for wsOIM°: ${{{\rm{w}}}}_{{{\rm{SO}}}}=\left\{\frac{8}{10},\frac{1}{10},\frac{1}{10}\right\}$, ${{{\rm{w}}}}_{{{\rm{OO}}}}=\left\{\frac{1}{10},\frac{8}{10},\frac{1}{10}\right\}$, ${{{\rm{w}}}}_{{{\rm{EVOO}}}}=\left\{\frac{1}{10},\frac{1}{10},\frac{8}{10}\right\}$, where ${{\rm{w}}}=\left\{{{{\rm{w}}}}_{{{\rm{SO}}}},{{{\rm{w}}}}_{{{\rm{OO}}}},{{{\rm{w}}}}_{{{\rm{EVOO}}}}\right\}$. To perform wsOIM*, the weighting scheme w^*^={0.07, 0.77, 0.16} was proposed based on the formula reported in Supplementary Method 4.

Fourthly, each bootstrap sample of consumers was merged with the array of bootstrap mean occurrences. DEs for an Upper Tail Percentile (UTP), the 95^th^ percentile, and their 95% CIs, were computed according to above.

## Results

**Fig. 3:**
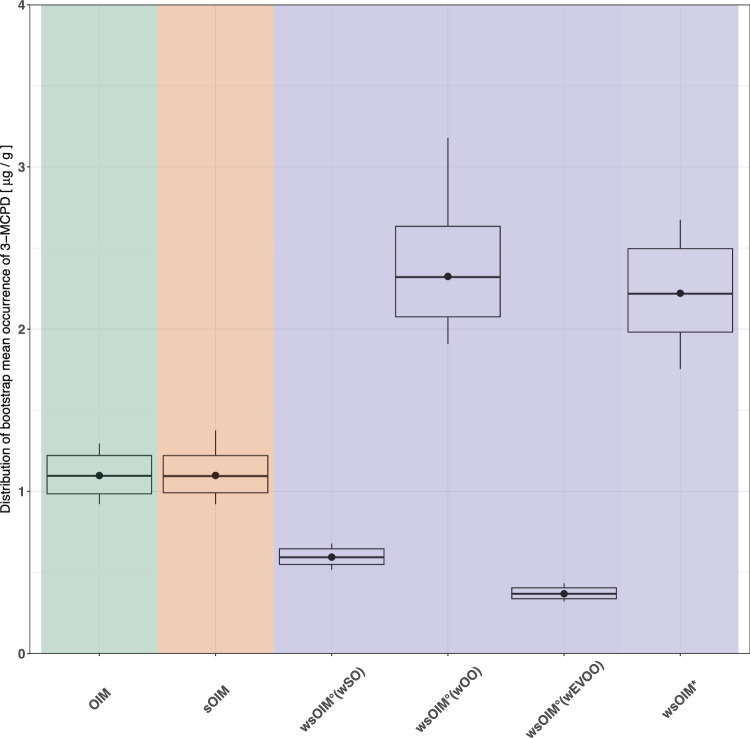
Distributions of bootstrap mean occurrence computed according to OIM, sOIM, wsOIM° and wsOIM*. The black points, the horizontal lines within the boxes and the lower and upper borders of the boxes respectively depict the means, the medians, the 2.5^th^ and the 97.5^th^ percentiles of these distributions. Each distribution is obtained from an equal number of bootstrap iterations (*n* = 1000).

**Fig. 4:**
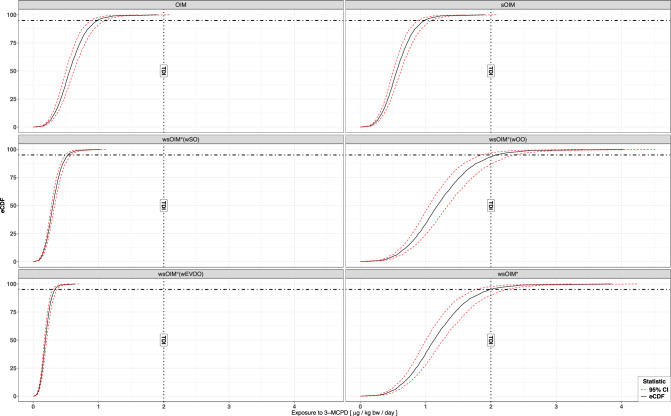
eCDFs of the distributions of exposure computed according to OIM, sOIM, wsOIM° and wsOIM*, and their 95% CIs. Each distribution is obtained from an equal number of bootstrap iterations (*n* = 1000). CI Confidence Interval, eCDF empirical Cumulative Distribution Function.

**Fig. 5:**
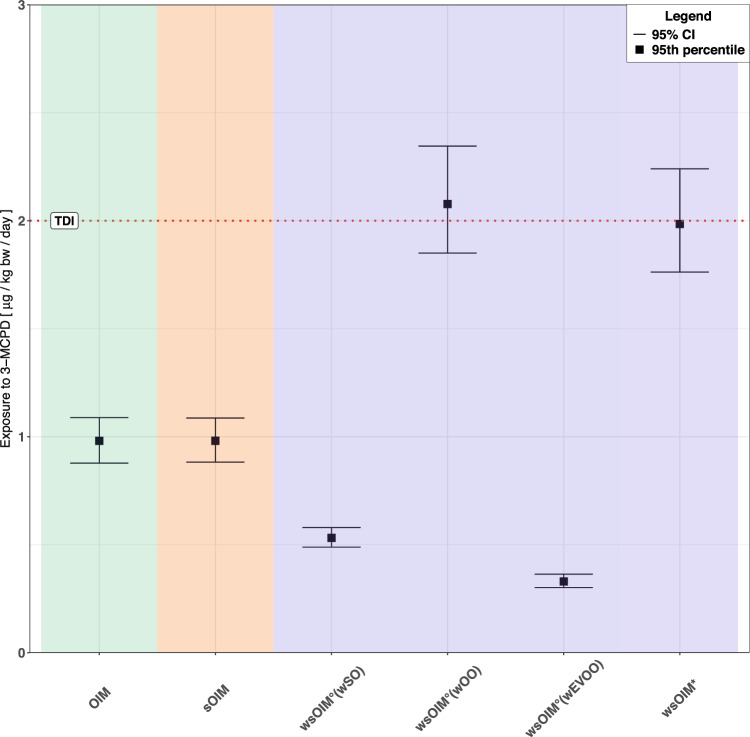
DEs for the 95^th^ percentile of the distributions of exposure computed according to OIM, sOIM, wsOIM° and wsOIM*, and their 95% CIs. Each distribution is obtained from an equal number of bootstrap iterations (*n* = 1000). CI Confidence Interval, DE Deterministic Estimate.

In the upcoming sections, comparisons across results obtained through the different methodologies are presented following an increasing degree of refinement of OIM. The methodologies were compared against each other based on different features. Firstly, the distributions of bootstrap mean occurrence (Fig. 3), the only varying feature across methodologies, were inspected. Secondly, their exposure estimates were compared (Fig. 4, Fig. 5). The deterministic eCDFs for exposure, and their 95% CIs, were depicted in Fig. 4. The UTPs, and their 95% CIs, were more closely visualized in Fig. 5 to carry out statistical testing.

### sOIM versus OIM

These methodologies were compared to investigate the effects of correcting for the violation of the i.i.d. assumption. As expected, the stratified bootstrap did not modify the position of the distribution of bootstrap mean occurrence, but only slightly narrowed its variability (Fig. 3). Such trend was reflected in similar DEs both for the eCDF and the UTP, but narrower CIs (Fig. 4, Fig. 5).

### wsOIM* versus OIM

The methodologies were compared to investigate the effects of correcting for the violation of the i.i.d. assumption and weighting by consumption. The distribution of bootstrap mean occurrence computed according to wsOIM* was higher and more variable than according to OIM (Fig. 3). Expectedly, the eCDF for wsOIM* was shifted towards higher values and the CIs were wider (Fig. 4). This was also the case for the UTP, which was observed to be not statistically different from the TDI at 0.05 significance level (Fig. 5).

### wsOIM* versus sOIM

The methodologies were compared to investigate the effects of weighting by consumption, given the correction for violation of i.i.d. In our case study, the comparison of these methodologies would lead to conclusions similar to those reported in the section “wsOIM* versus OIM”, given that the distribution of bootstrap mean occurrence according to sOIM is similar to the one computed according to OIM.

### wsOIM° versus sOIM

The methodologies were compared to investigate the robustness of the assumption of equal weighting. The distributions of bootstrap mean occurrence for the proposed wsOIM° scenarios were shifted towards lower (w_EVOO_, w_OO_) or higher (w_SO_) values compared to OIM. Their variability decreased (w_SO_, w_EVOO_) or increased (w_OO_) compared to OIM (Fig. 3). These trends were reflected in a shift of eCDFs towards higher (w_EVOO_, w_OO_) or lower (w_SO_) values, with CIs being narrowed (w_SO_, w_EVOO_) or widened (w_OO_), as shown in Fig. 4. Notably, the UTP for w_OO_ was not statistically different from the TDI at 0.05 significance level (Fig. 5).

### wsOIM* versus wsOIM°

The methodologies were compared to investigate the robustness of weighting by consumption. The bootstrap mean occurrence computed through wsOIM* was not different from wsOIM°(w_OO_) and different from wsOIM°(w_SO_) and wsOIM°(w_EVOO_) at 0.05 significance level (Fig. 3). The resulting eCDF for wsOIM* pointed to higher values than for wsOIM°(w_SO_), wsOIM°(w_EVOO_) and to lower values than for wsOIM°(w_OO_). UTP for wsOIM* was not statistically different from the one computed according to wsOIM°(w_OO_) at 0.05 significance level.

## Discussion

### Risk characterization

The findings of our case study can be used for risk characterization, with the caveat that only vegetable oils contributed to exposure to 3-MCPD, and occurrence data were simulated. Therefore, realistic comparison with the TDI cannot be adequately addressed. The following risk characterization was inspired by the Tipping Point methodology [20].

Correcting for i.i.d did not meaningfully influence risk characterisation, as the UTP computed according to OIM was already statistically different (in this case, lower) from the TDI. However, additional adjustment for consumption led to re-examination of this conclusion, as the UTP computed according to wsOIM* was not statistically different from the TDI.

Nonetheless, the robustness of these conclusions was investigated by wsOIM°. We regarded the assumptions as robust if any of the estimates for UTP computed according to wsOIM° was pointing to higher risk than in the selected methodology, i.e. equality/exceedance of the TDI not already shown in the selected methodology. Investigation of the assumption of equal weighting was compelling, as the estimate computed according to sOIM pointed to non-exceedance of the TDI. This assumption was not robust. On the other hand, weighting for consumption was robust, as the estimate according to wsOIM* already pointed to no statistical difference from the TDI. Altogether, the robustness of our risk assessment would have been affected by the application of such methodologies.

In conclusion, with respect to the implementation of sOIM and wsOIM* in real-world exposure assessments, the accuracy of risk assessments should not suffer from biases introduced by oversimplification of assumptions, since setting too low TDIs may lead to lack of effective regulatory measures, and setting too high TDIs may lead to implementation of unnecessary, costly interventions to reduce the degree of exposure to the contaminant. This is also why the robustness of the adjustments should be assessed by wsOIM°. Our case study aims to prompt the scientific community to ponder these aspects in exposure assessments.

### Summary

In this article, we devised and demonstrated statistical methodologies based on OIM. Their results are easily interpretable as they employ basic statistical concepts. The expected degree of violation of the assumptions and the availability of raw consumption and occurrence data should drive the choice of the methodologies to be implemented after performing OIM. If employed as standalone techniques, a tiered approach should be considered, although they all require seamless implementation and negligible computational time. Nonetheless, the stratified bootstrap should not be employed to compensate for inadequate sample size within subcategories. Among the presented methodologies, the preferred choice for risk assessors should be wsOIM* if violation of both assumptions is suspected, and detailed consumption is available. Nonetheless, sOIM should suffice if only the i.i.d. assumption is expected to be undermined and the results of wsOIM* not to differ from sOIM. In cases where the consumption data cannot reliably guide the generation of weights, wsOIM° could be implemented subsequently to wsOIM* to explore the robustness of the weighting scheme. In any case, wsOIM° could be implemented subsequently to sOIM and wsOIM* to explore their robustness. However, among the presented methodologies, wsOIM° is the most computationally demanding. Moreover, wsOIM° may be regarded as the least interpretable one, as expert support becomes essential to design the weighting schemes, which should depend on culture-specific dietary habits.

### Future perspectives

sOIM and wsOIM are suitable for application on occurrence data derived from large, open-access occurrence datasets, such as the Information Platform for Chemical Monitoring [27]. In these cases, application of wsOIM* becomes of particular interest as it could adjust for the relevance of each collected occurrence sample in a real-world scenario, and for the type of sampling design used for their collection.

wsOIM* can also support inclusion of information on market share, which are, however, rarely publicly available.

It should be investigated whether our methodologies affect the overestimation of UTPs compared to more complex methodologies not relying on OIM.

In conclusion, throughout this paper, modifications of a methodology for dietary PEA were discussed. These may allow governmental agencies performing risk management to base their regulatory decision-making on more informative and comprehensive methodologies.

## Supplementary information

Supplementary information

## Data Availability

The consumption datasets C1 (“consumption_user.csv”) and C2 (“subject_user.csv”) are publicly available at FAO/WHO GIFT repository after sign-in (https://www.fao.org/gift-individual-food-consumption/en; accessed on September 5^th^, 2022). The occurrence dataset used for the simulation and the relevant R code are available on Zenodo (https://zenodo.org/communities/irfmn-irccs/?page=1&size=20).
