# Supplementary material for: Refined methodologies for probabilistic dietary exposure assessment for food contaminants based on the observed individual means methodology
Source: J Expo Sci Environ Epidemiol. 2025 Jan 29;35(3):375–81. doi: 10.1038/s41370-024-00740-4 (PMC12069084; doi:10.1038/s41370-024-00740-4)
Supplement: Supplementary file 1 — Supplementary information [file 41370_2024_740_MOESM1_ESM.docx]

Supplementary Methods 1

Preliminary notation

Let $n$ be the sample size of the whole sample of consumers or of a subsample of interest.

Let $L$be the categorical variable identifying categories and let $A$ be the number of categories selected for Probabilistic Exposure Assessment (PEA), so that:

$L=\{l_{1},\ldots,l_{a},\ldots,l_{A}\}$

( 1 )

Let *DCONS* be the quantitative, semipositive variable identifying daily consumption observed on occasion of a dietary survey quantitatively reporting daily consumption of consumed food, so that ${dcons}_{l_{a},i}$ indexes the daily consumption of food belonging to category $l_{a}$ for individual $i,$ $\forall a,\forall i = \{1, \ldots$ *, n}*. Let *BW* be the variable identifying body weight for the individuals, so that $bw_{i}$ indexes the body weight of individual $i,$ $\forall i$.

In the framework of sampling only contaminated food products belonging to categories observed to be consumed in the dietary survey, let $K\left( l_{a} \right)$ be the number of analysed food samples within category $l_{a}$. Occurrence values $C$ may be indexed as follows, whether above or under Limit Of Detection (LOD):

$$C=\left\{ c_{l_{1},1},\ldots,c_{l_{a},k\left( l_{a} \right)},\ldots,c_{l_{A},K\left( l_{A} \right)} \right\}$$

( 2 )

For each category, arithmetic mean occurrence $\overline{c}_{l_{a}}$can be computed.

The above-reported notation is sufficient to build Observed Individual Means (OIM). Nonetheless, the following additional notation has to be introduced for stratified Observed Individual Means (sOIM) and weighted stratified Observed Individual Means (wsOIM).

Let $M$ be the categorical variable identifying subcategories and let $B(a)\mid a$ be the number of subcategories within category $l_{a}$, so that, $\forall a$:

$$M\mid l_{a}=\left\{ m_{1\mid a},\ldots,m_{b\left( a \right)\mid a},\ldots,m_{B\left( a \right)\mid a} \right\}$$

( 3 )

Let $K\left( l_{a},m_{b\left( a \right)\mid a} \right)$ be the number of samples, within subcategory $m_{b\left( a \right)\mid a}$, for which information on occurrence is provided. Occurrence values may now be indexed as follows:

$$C=\left\{ c_{l_{1},m_{1},1},\ldots,c_{l_{a},m_{b\left( a \right)\mid a},k\left( l_{a},m_{b\left( a \right)\mid a} \right)},\ldots,c_{l_{A},m_{B\left( A \right)\mid A},K\left( l_{A},m_{B\left( A \right)\mid A} \right)} \right\}$$

( 4 )

Computation of Deterministic Estimates

In general, chronic exposure to food contaminants *EXPO* is computed as function of occurrence, consumption and body weight.

First, category-specific exposure for each individual is computed:

$$expo_{l_{a},i}=\frac{dcons_{l_{a},i}\times\overline{c}_{l_{a}}}{bw_{i}}$$

( 5 )

Secondly, individual exposure is computed:

$$expo_{\cdot,i}= \sum_{a=1}^{A} expo_{l_{a},i}$$

( 6 )

obtaining a distribution for exposure.

Thirdly, the inverse Cumulative Distribution Function (iCDF) is employed to compute the value of exposure corresponding to a predetermined percentile of interest (ex: the 95^th^ percentile): a deterministic estimate is computed.

Supplementary Methods 2

Computation of Probabilistic Estimates (OIM)

Let $S$ be the number of iterations of the algorithm (simulations). PEs are obtained as follows:

1. Application of non-parametric bootstrap to consumption dataset results in a subset of consumers for each iteration of the algorithm. For each simulation $s$, a set of $n^{*}=n$ individuals are resampled with replacement from the dietary survey dataset:
   - For each resampled individual $i^{*}$, information about body weight is retained; this can be indexed as $bw_{s,i^{*}}$;
   - For each resampled individual $i^{*}$, for each category $l_{a}$, information about daily consumption is retained; this can be indexed as $dcons_{s,l_{a},i^{*}}$;
2. On the other hand, separate application of non-parametric bootstrap to occurrence dataset results in a value of bootstrap arithmetic mean occurrence. For each simulation $s$, for each category $l_{a}$, $K^{*}\left( l_{a} \right)=K\left( l_{a} \right)$ occurrence values are resampled with replacement from the occurrence dataset and their bootstrap arithmetic mean occurrence ${\overline{c}^{*}}_{s,l_{a}}$ is computed and retained;
3. Subsequently, bootstrapped data on occurrence, daily consumption and body weight are merged by index of simulation $s$, resampled individual $i^{*}$ and category $l_{a}$ to obtain a category-specific estimate for exposure:

$expo_{s,l_{a},i^{*}}=\frac{dcons_{s,l_{a},i^{*}}\times{\overline{c}^{*}}_{s,l_{a}}}{bw_{s,i^{*}}}$, $\forall s, \forall a, \forall i^{*}$

( 7 )

1. Overall exposure for each resampled individual is computed as

$expo_{s, \cdot,i^{*}}= \sum_{a=1}^{A} expo_{s,l_{a},i^{*}}$, $\forall s, \forall i^{*}$

( 8 )

For each simulation $s$, a random distribution for exposure is obtained.

1. For each simulation $s$, iCDF is employed to compute the value of exposure corresponding to a predetermined percentile of interest (ex: the 95th percentile), therefore, considering the entirety of the simulations, a distribution of $S$bootstrapped values is obtained. Given a confidence level $100(1-\alpha)\%$, using the percentile method, the $\frac{\alpha}{2}$ and $1-\frac{\alpha}{2}$ quantiles of this distribution represent the probabilistic estimates for the corresponding deterministic estimate. The width of such interval indicates the degree of uncertainty related to the statistic. Other statistics computed from this distribution may be of general interest in the probabilistic exposure estimation process, for example in assessing the skewness of the distribution of bootstrapped values.

**Supplementary Methods 3**

**Goodness of fit for experimental data on occurrence (subcategory Seed Oils)**

| **Distribution** | **Akaike Information Criterion** |
| --- | --- |
| Normal | 25.11 |
| Lognormal | 14.79 |
| Gamma | 14.96 |
| Weibull | 14.87 |

Supplementary Methods 4

Set of weights for wsOIM*

Given that in our case daily consumption could also be indexed as ${dcons}_{l_{a},m_{b\left( a \right)\mid a},i}$ because additional information about subcategory was available in consumption dataset *C1*, the consumption-aware vector of weights for wsOIM* was computed as follows:

$w_{j}^{*}=\frac{\sum_{i=1}^{N} dcons_{VO,j,i}}{\sum_{\left( b\left( a \right) | a \right)=1}^{B\left( a \right)} \sum_{i=1}^{N} dcons_{VO,j,i}}$, $j= \{SO, OO, EVOO\}$

( 9 )
